# The effect of TGF-β_1_ polymorphisms on pulmonary disease progression in patients with cystic fibrosis

**DOI:** 10.1186/s12890-022-01977-1

**Published:** 2022-05-07

**Authors:** T. Trojan, Miguel A. Alejandre Alcazar, G. Fink, J. C. Thomassen, M. V. Maessenhausen, E. Rietschel, P. M. Schneider, S. van Koningsbruggen-Rietschel

**Affiliations:** 1 CF Centre Cologne, Children’s Hospital, Faculty of Medicine and University of Cologne, Kerpener Str. 62, 50937 Cologne, Germany; 2 Translational Experimental Pediatrics - Experimental Pulmonology, Department of Pediatric and Adolescent Medicine, Center for Molecular Medicine Cologne (CMMC), and Cologne Excellence Cluster for Stress Responses in Ageing-Associated Diseases (CECAD), Faculty of Medicine and University Hospital Cologne, University of Cologne, Cologne, Germany; 3 Institute for Lung Health (ILH), University of Giessen and Marburg Lung Center (UGMLC), Member of the German Center for Lung Research (DZL), Giessen, Germany; 4 Institute of Legal Medicine, Faculty of Medicine and University Hospital, University of Cologne, Cologne, Germany

**Keywords:** Transforming-growth-factor Beta 1 (TGF-β_1_), Cystic fibrosis, Polymorphisms, Inflammation, Forced expiratory volume in one second (FEV_1_), *Pseudomonas aeruginosa*

## Abstract

**Background:**

Transforming Growth Factor-β_1_ (TGF-β_1_) is a genetic modifier in patients with cystic fibrosis (CF). Several single nucleotide polymorphisms (SNPs) of TGF-β_1_ are associated with neutrophilic inflammation, lung fibrosis and loss of pulmonary function.

**Aim:**

The aim of this study was to assess the relationship between genetic TGF-β_1_ polymorphisms and pulmonary disease progression in CF patients. Furthermore, the effect of TGF-β_1_ polymorphisms on inflammatory cytokines in sputum was investigated.

**Methods:**

56 CF-patients and 62 controls were genotyped for three relevant SNPs in their TGF-β_1_ sequence using the SNaPshot® technique. Individual “slopes” in forced expiratory volume in 1 s (FEV_1_) for all patients were calculated by using documented lung function values of the previous five years. The status of *Pseudomonas aeruginosa (Pa)* infection was determined. Sputum concentrations of the protease elastase, the serine protease inhibitor elafin and the cytokines IL-1β, IL-8, IL-6, TNF-α were measured after a standardized sputum induction and processing.

**Results:**

The homozygous TT genotype at codon 10 was associated with a lower rate of chronic *Pa* infection (*p* < 0.05). The heterozygous GC genotype at codon 25 was associated with lower lung function decline (*p* < 0.05). Patients with homozygous TT genotype at the promotor SNP showed higher levels of TNF-α (*p* < 0,05). Higher levels of TGF-β_1_ in plasma were associated with a more rapid FEV_1_ decline over five years (*p* < 0.05).

**Conclusions:**

Our results suggest that polymorphisms in the TGF-β_1_ gene have an effect on lung function decline, *Pa* infection as well as levels of inflammatory cytokines. Genotyping these polymorphisms could potentially be used to identify CF patients with higher risk of disease progression. TGF-β_1_ inhibition could potentially be developed as a new therapeutic option to modulate CF lung disease.

**Supplementary Information:**

The online version contains supplementary material available at 10.1186/s12890-022-01977-1.

## Background

Cystic fibrosis (CF) is an autosomal recessive, genetic disorder that affects approximately 85,000 individuals worldwide [1]. This multisystemic disorder is caused by mutations affecting the Cystic Fibrosis Transmembrane Conductance Regulator (CFTR) in the epithelial membrane of exocrine glands, which lead to dysfunctional fluid and ion-transport causing a production of thickened mucus [2]. Pathological mucociliary clearance leads to CF lung disease, which over time becomes the major life-shortening factor for CF patients [1]. Chronic plugging of bronchioles with secretions, recurring bacterial infections and pulmonary exacerbations instigate the development and retention of a hostile inflammatory environment in the lungs, leading to tissue breakdown and irreversible lung damage [3]. The most relevant microorganism in CF lungs, *Pseudomonas aeruginosa* (*Pa*), provokes a vigorous inflammatory response with neutrophilic infiltration of airways and subsequent damage by the release of proteases and oxidants [4]. This dysregulated chronic state of inflammation in CF airways is sustained by a variety of proinflammatory mediators including TNF-α, IL-1β, IL-6 and IL-8 and leads to a decline in lung function caused by bronchiectasis and irreversible fibrotic remodeling of lung tissue [4]. 98% of all CF patients die of progressive respiratory insufficiency [5].

Whilst prevalent CFTR mutations are an important determinator for the severity of CF lung disease, the genotype–phenotype correlation between the genetically determined loss of CFTR function and lung function decline is approximately 60% [6, 7]. This suggests that other non-CFTR related factors, such as genetic modifiers with a regulatory effect on the inflammatory response in CF lungs, may also have a significant impact on lung function decline in CF patients.

TGF-β_1_ has been identified as such a genetic modifier for CF lung disease [6]. Produced by bronchial epithelial cell, this growth factor acts with a localized, modulatory role in the recruitment and activation of neutrophilic granulocytes within a complex network of inflammatory and anti-inflammatory cytokines, thereby regulating inflammatory processes, specifically in context of chronic pulmonary disease [8]. TGF-β_1_ inhibits the degradation of extracellular matrix by stimulating protease-inhibitors leading to fibrotic reconstruction of lung tissue [9]. Furthermore, it promotes smooth muscle cell hypertrophy and hyperplasia [4, 10].

In a recent study, Sagwal et al. have shown that levels of serum TGF-β_1_ were increased in pulmonary exacerbation phases, in infection with *Pa* and in subjects with a ΔF508 mutation [11]. TGF-β_1_ levels decreased significantly after antibiotic treatment of pulmonary exacerbations [11].

Moreover, it has been shown that TGF-β_1_ has an inhibitory effect on the biogenesis of CFTR and prevents the functional rescue of delF508-CFTR [10]. In a recent study by Mitash et al., TGF-β_1_ has been associated with degradation of CFTR mRNA in human bronchial epithelial cells via recruitment of microRNAs to an RNA-induced silencing complex [12]. Snodgrass et al. have shown that TGF-β_1_ was associated with CFTR inhibition and prevention of functional rescue in human epithelial cells [10].

However, in vivo levels of TGF-β_1_ are dependent on specific polymorphisms in the TGF-β_1_ gene [13]. So far, few studies have investigated the effects of genetic polymorphisms of TGF-β_1_ on lung function. In the context of CF, three single nucleotide polymorphisms (= SNPs) have previously been investigated. Each of these polymorphisms, i.e. rs1800469 located in the promotor region and both rs1800470 and rs1800471 located in Exon 1 of the TGF-β_1_ gene, result in a change in the primary amino acid sequence of the TGF-β_1_ [6, 13, 14].

In previous studies it was shown that some of these TGF-β_1_ polymorphic genotypes are associated with higher TGF-β_1_ expression, a steeper decline in pulmonary function (FEV_1_) as well as increased pulmonary fibrosis [6, 13, 15, 16]. However, some of the results among these studies are contradictory, as different genotypes were associated with a decrease in pulmonary function and worse clinical status. Furthermore, very little is known about the impact of a TGF-β_1_ polymorphism-related dysregulation of the signal pathway of TGF-β_1_ on the complex inflammatory response of the CF airways. It has to be noted, however, that immunological factors contributing to or perhaps even enabling the onset of bacterial infection with *Pa*, one of the major predictors for mortality and morbidity for CF patients, could not yet be identified [17].

The primary aim of this study was to investigate whether TGF-β_1_ SNP genotypes, as modifiers of CF lung disease, can be associated with a faster decline in pulmonary function. To our knowledge, there is no data correlating TGF-β_1_ phenotypes with the individual FEV_1_ slopes of CF patients. FEV_1_ correlates with morbidity and mortality of CF-patients and is a gold standard outcome parameter in routine diagnostics to assess disease progression as well as in clinical studies to investigate the efficacy of new drugs [18]. Furthermore, we wanted to investigate whether TGF-β_1_ polymorphisms are associated with higher TGF-β_1_ expression, higher *Pa* infection rates and elevated levels of proinflammatory cytokines in sputum.

## Materials and methods

### Study population

The TGF-β_1_ genotypes for all three SNPs were determined in 56 CF-patients and 62 healthy controls. All CF patients had a confirmed diagnosis of CF according to the consensus guidelines of the Cystic Fibrosis Foundation [19]. Inclusion criteria were a signed informed consent and the ability of patients to expectorate sputum. Exocrine pancreatic insufficiency was diagnosed by repeated pancreas elastase testing of patients´ stools and confirmed by repeated levels < 200ug/g. The genomic DNA of CF patients was isolated from a whole blood sample obtained during the routine yearly blood sample collection. The DNA samples of 62 randomly chosen, healthy controls were obtained from paternity test samples at the Institute of Legal Medicine, to which the individuals had given their consent when these samples were obtained [20]. Patients with an acute pulmonary exacerbation at the study visit were excluded. All pulmonary function tests performed during the 5-year interval between 2010 and 2014 were reviewed. The best FEV_1_ value for every year was obtained for FEV_1_ slope calculation. 15 patients underwent changes in CF therapy (e.g. start of CFTR-modulatory therapy) or lung transplantation within this period of time. For these, a different five-year time span, prior to their new therapy, was chosen for calculation of the individual FEV_1_ slope.

### *TGF-β*_*1*_* polymorphism genotyping*

DNA was extracted from whole blood and diluted to a standard concentration of 1 ng/$\mu$ l. The DNA was then amplified using Polymerase Chain Reaction (PCR) with specific primers designed to amplify two separate targets of the genome, containing the relevant SNPs, using *Primer3Plus, BLAST and NCBI Electronic PCR-Software* [21, 22]. Primers were produced and shipped by *biomers.net* [23]. PCR primer sequences used are available on request. Agarose gel electrophoresis tests of the amplicons were performed to monitor the correct amplification of the two targets. Enzymatic purification of samples followed using Exonuclease and Shrimp Alkaline Phosphate (SAP). According to instructions of the SNaPshot™ Multiplex Kit (*Applied Biosystems*), a Single Base Extension (SBE) with didesoxyribonucleosid-triphosphates (ddNTPs), marked with four different fluorescent signals, QIAGEN Mastermix (containing DNA Polymerase AmpliTaq©, reaction puffer) was performed in a thermocycler (Gene AMP PCR System 2720 thermocycler, *Applied Biosystems*) [24–26]. SNP typing primer sequences were GGCAACAGGACACCTGA(A/G) for SNP rs1800469, CAGCGGTAGCAGGAGC(G/A) for Codon 10 SNP rs1800470 and GTGCTGACGCCTGGCC(G/C) for Codon 25 rs1800471.

Lastly, after enzymatic purification of the Single Base Extension reaction (SBE) reaction products, capillary electrophoresis (using *ABI Prism 3130 Genetic Analyzers)* was performed to determine the genotype of each SNP for all patients and controls using the software *Genemapper 4.0* (*Applied Biosystems*). An exemplary capillary electrophoresis result of one patient’s genotype for all three polymorphisms is shown in Additional file 1: Figure A. Materials, concentrations, PCR primers sequences and exact reaction conditions for PCR and Single Base Extension (SBE) are available on request.

### Spirometry

Spirometric measurements were performed according to the ATS guidelines using GLI references and assessed before any other study assessment with Master Screen Body (Jaeger, Heidelberg, Germany) and SentrySuite™ version 2.19 software (Carefusion, Becton Dickinson, Franklin Lakes, New Jersey, USA) [27]. For each measurement, the best FEV_1_ value was used for analysis. The best *yearly* FEV_1_ value was used in a linear regression model to calculate individual FEV_1_ slope values for every patient. Patient results were also analyzed within different FEV_1_ subgroups and FEV_1_ slope subgroups, according to FEV_1_ progression over time.

### Pseudomonas aeruginosa (Pa) infection

The status of *Pa* infection, defined by clinically established Leeds criteria, was obtained from the patients’ files and is described according to the following three groups: *Pa* positive (= chronic infection), *Pa* naïve (= never infected) or *Pa* negative (infected in the past, currently not infected after eradication therapy) [28].

### *Sputum analysis of TGF-β*_*1*_* and other cytokines*

As part of the regular outpatient visits, patients induced their sputum by inhalation of hypertonic saline during a routine physiotherapist session. This sputum was processed according to the standard operating procedure (SOP) of the TDN (Therapeutic Drug Development Network, USA). Concentrations of elastase and elafin in sputum were determined by specific ELISA assays (EnzChek® Elastase Assay Kit,—Molecular Probes Europe, Leiden, Netherlands; Elafin/Skalp Human ELISA Kit—abcam, Cambridge, UK). Pro-inflammatory cytokine concentrations in sputum were assessed using a human inflammatory cytokine ELISA-kit (BD Cytometric Bead Array Humane Inflammatory Cytokine Kit, San Jose, CA, USA). TGF-β_1_ levels in sputum and plasma were determined by a TGF- β_1_ specific ELISA-kit (Quantikine®ELISA Human TGF-β_1_, R&D systems, Minneapolis, MN, USA).

### Statistical analysis

IBM SPSS Statistics 24 was used for statistical analysis. To compare two metric variables, we correlated using Pearson’s test. For correlation between one metric and one discontinuous variable, we used the Kruskal–Wallis test. For tests correlating two discontinuous variables we used cross-classified tables with exact Fisher’s test. For all tests, a p-value < 0,05 was considered statistically significant.

For a detailed analysis of FEV_1_ slopes, different patient subgroups were formed as summarized in Additional file 1: Figure B. One categorization involved a comparison between patients with positive FEV_1_ slope and those with negative slopes (Categorization 1). Two further categorizations were used to compare patients with steepest decline in FEV_1_ to patients with a relatively steady FEV_1_ (with only little decline or even small improvements) and patients with clear improvements in FEV_1_ over the period of investigation (Categorization 2 & 3). These categories were formed to investigate inflammatory status in different stages of CF lung disease and to determine the role of TGF-β_1_ in this process.

Additionally, for statistical analysis, patient subgroups were also formed according to patients’ absolute, best FEV_1_ at the end of the observed 5 year-period. Here patients were analyzed in different FEV_1_ subgroups in order to investigate patients who finished their 5-year FEV_1_ slope in a “normal” FEV_1_ group (> 80% predicted), an “intermediate” FEV_1_ group (40–80% predicted) or a “low” FEV_1_ group (< 40% predicted). A summary of these subgroups can be found in Additional file 1: Figure C.

For more detailed analysis of TGF-β_1_ genotypes, for some statistical investigations we used subgroups of combined genotypes to explore the impact of a heterozygous genotype when compared to homozygous genotypes (e.g. CT vs TT/CC).

## Results

### Study population

The mean age of CF-patients was 21 years (SD ± 11.1 years). The mean FEV_1_ at the time of blood sampling was 74.26% predicted (SD ± 25.36% predicted). The mean FEV_1_ slope of patients was -1.81%FEV_1_ change per year (SD ± 3.20%FEV_1_ change). 33.9% of patients were chronically infected with *Pa*. In our CF cohort, 29 patients (approx. 52%) were F508del homozygous, whilst 21 patients (approx. 37%) were F508del heterozygous and 6 patients (approx. 11%) carried two other CF-causing mutations. Furthermore, 53 patients (approx. 95%) in our cohort showed exocrine pancreatic insufficiency.

The demographic results of our cohort are presented in Table 1.

**Table 1:** Clinical data of CF patients

Parameter	Mean
Age (years)	21 (SD ± 11.1)
Adult: Children ratio (%)	57:43
Sex ratio (m: f) (%)	55:45
FEV_1_ (%predicted)	74.26 (SD ± 25.36)
FEV_1_ slope (%FEV_1_ change/year)	− 1.81 (SD ± 3.20)
Prevalence of chronic *Pa* infection (%)	33.90
Prevalence of pancreatic-insufficiency (%)	95
Ratio F508del homozygous: F508del heterozygous: other CF Mutations (%)	52: 37: 11

As shown in Table 2, there was no significant difference in the distribution of genotypes or alleles in the investigated cohort of 56 CF patients compared to 62 healthy controls. The genotype results were successfully tested for conforming to expected distributions according to the Hardy Weinberg Equilibrium.

**Table 2:** Distribution of genotypes in CF-patients and controls

Genotypes	*CF patients*		*Controls*	
	n	*%*	n*	%
*Promotor*				
CC	20	35.7	26	44.1
CT	31	55.4	28	47.5
TT	5	8.9	5	8.5
*Codon 10*				
CC	7	12.5	9	14.8
CT	31	55.4	32	52.5
TT	18	32.1	20	32.8
*Codon 25*				
GG	50	89.3	55	88.7
GC	6	10.7	6	9.7
CC	0	0	1	1.6

^*^As shown in Table 2, in our control group, the total “n” for Promotor and Codon 10 genotype results were 59 and 61 respectively, due to unclear technical problems in genotyping of singular SNPs of 3 and 1 control samples, respectively

### *TGF- β*_*1*_* polymorphisms and pulmonary status*

#### Codon 10

**Fig. 1:**
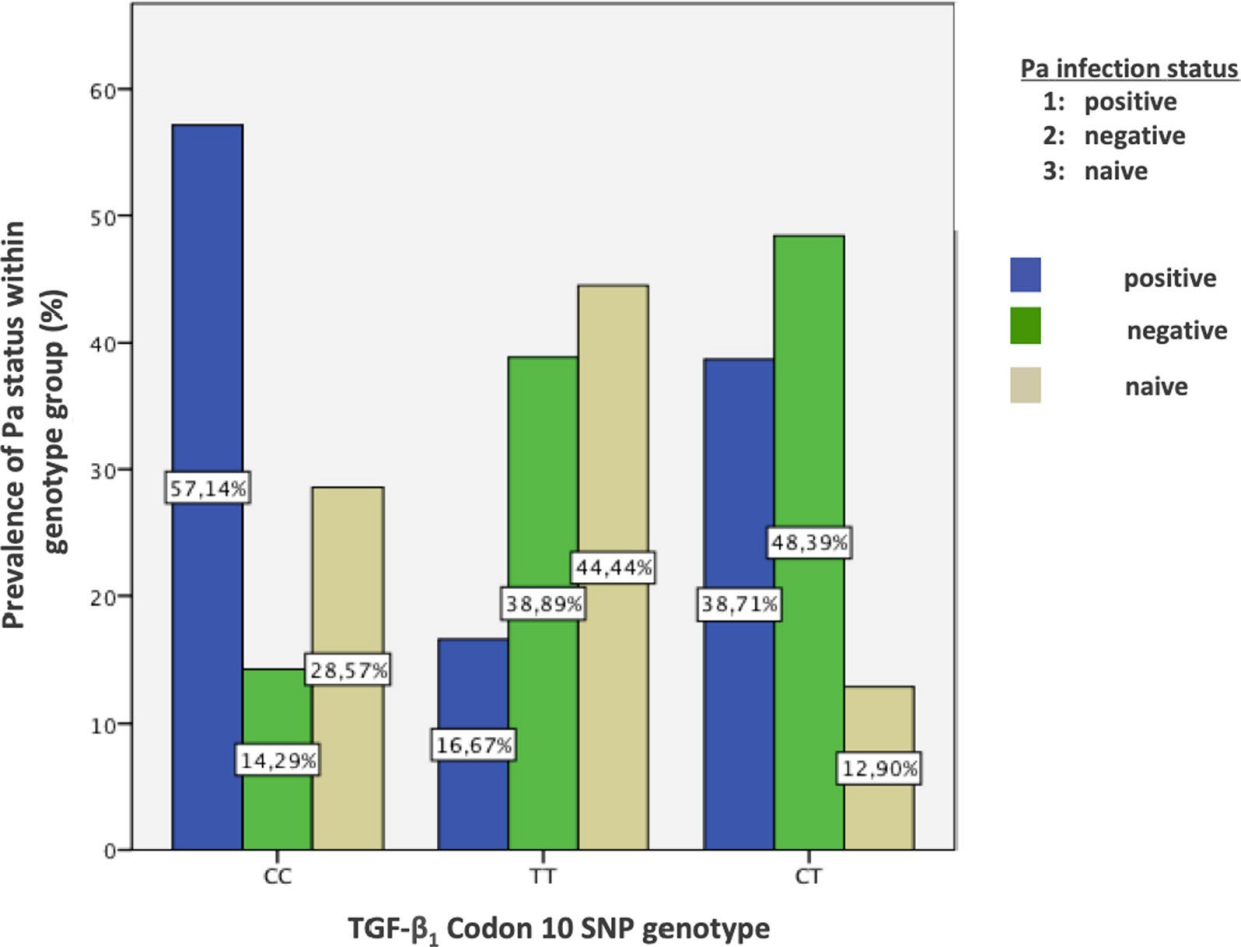
TGF- β_1_ codon 10 SNP genotypes and prevalence of *Pa* infection status within the genotype group

The homozygous TT genotype at codon 10 was significantly associated with a lower *Pa* infection rate, as demonstrated in Fig. 1. 16.7% of patients with this genotype were infected with *Pa*, compared to 42.1% in the combined CC/CT genotype group (p = 0.047). Of all patients with chronic *Pa* infection in our cohort (n = 19), 15.2% showed the TT genotype. No significant associations between the FEV_1_ slope or FEV_1_ slope subgroups and genetic polymorphisms at codon 10 were found.

#### Codon 25

For the polymorphism at codon 25, we found no significant association with *Pa* infection rate. However, we were able to show that the group of patients with the heterozygous GC genotype contained a significantly higher proportion of patients with positive FEV_1_ slopes, with positive FEV_1_ change greater than 1.0% per year (p = 0.003) according to FEV_1_ slope categorization 2. As shown in Table 3, of all patients with the GC genotype, 66.7%, exhibited an FEV_1_ slope greater than + 1.0% FEV_1_ change/year, compared to only 10.2% of patients with a GG genotype. Baseline FEV_1_ in the GC-group was 72,7%, compared to 84,1% in the GG group. Also, according to FEV_1_ slope categorization 2, 100% of patients with stable FEV_1_ slopes (between -1%/year and + 1%/year) and 93% of the patients with declining FEV_1_ slopes (< -1%/year) presented with a GG genotype at codon 25.

**Table 3:** Prevalence of FEV_1_-slopes >  + 1%/year for patients with different Codon 25 genotypes

Genotype codon 25	Prevalence in FEV_1_-Slope Group > 1.0% FEV_1_ change/year		Mean Baseline FEV_1_ (in % predicted)
	n	%	
GC	6	66.7	72.7
GG	50	10.2	84.1

No significant associations of Codon 25 polymorphisms with absolute FEV_1,_ FEV_1_ slope or FEV_1_ slope subgroups were found in our cohort.

#### Promotor

We did not find a significant association between polymorphisms in the TGF-β_1_ promotor and patients’ *Pa* infection status, their FEV_1_ slopes or their FEV_1_ slope subgroups.

A summary of mean average FEV_1_ slope results for different SNP groups is shown in Additional file 1: Figure D.

### *TGF-β*_*1*_* concentration and FEV*_*1*_* slopes*

**Fig. 2:**
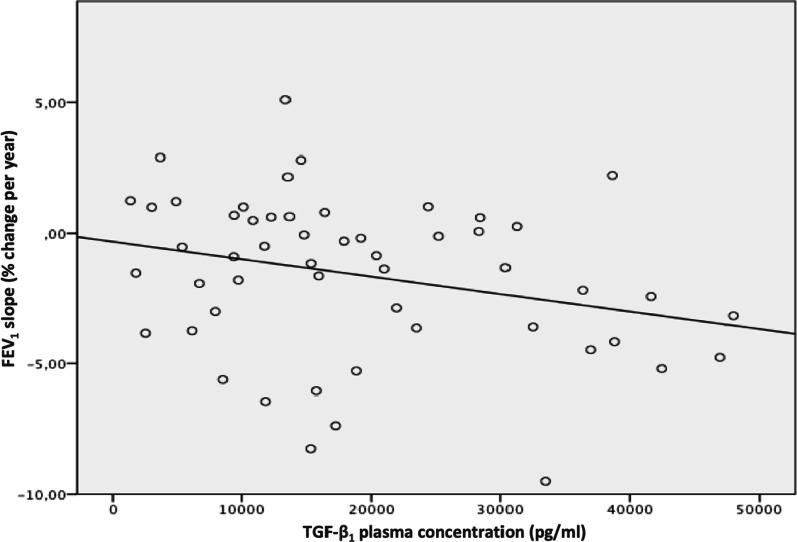
TGF- β_1_ plasma concentration and FEV_1_ slope of CF patients

Higher TGF-β_1_ concentrations in patients’ plasma significantly correlated with a steeper decline in FEV_1_ slope (p = 0.045). This is demonstrated in Fig. 2. Patients with FEV_1_ slopes that were below than -2%/year (Categorization 3) showed a higher TGF-β_1_ concentration (25,332 pg/ml) in plasma compared to patients with a positive FEV_1_ slope of greater than + 2%/year (16,754 pg/ml).

No correlation between TGF-β_1_ levels in plasma and FEV_1_ slope subgroups was found.

In our study, a higher concentration of TGF-β_1_ in the patients’ sputum significantly correlated with a positive FEV_1_ slope (> 0% FEV_1_ change/year) according to slope categorization 1 (p = 0.010). The median TGF-β_1_ concentration in sputum was 66.0 pg/ml higher in patients with a positive FEV_1_ slope (> 0%/year) compared to patients with a negative FEV_1_ slope (< 0%/year). TGF-β_1_ sputum levels showed no significant associations with other investigated FEV_1_ slope categorizations.

**Fig. 3:**
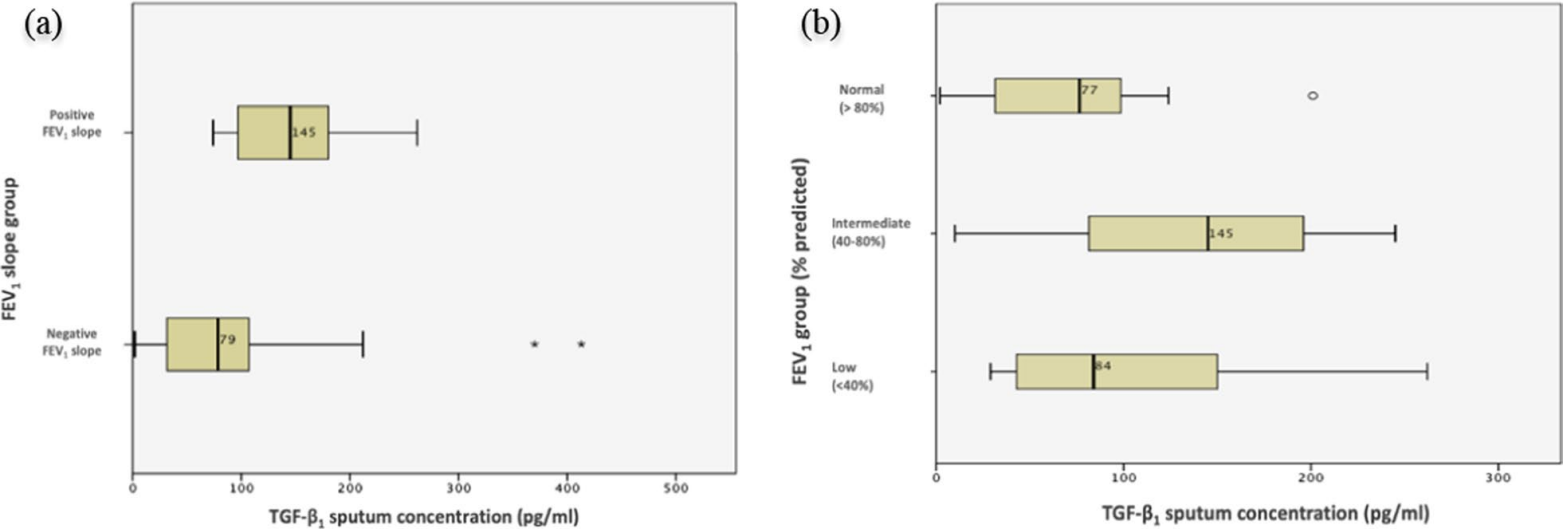
**a** TGF- β_1_ sputum concentration and FEV_1_ slope group, **b** TGF- β_1_ sputum concentration and FEV_1_ group over five years

However, patients with intermediate FEV_1_ values (40–80% predicted) at the end of their five year observation period, showed significantly higher concentrations of TGF-β_1_ in sputum compared to patients with normal (> 80%) or low (< 40%) FEV_1_ values (p = 0.01). These results are demonstrated in Fig. 3a, b.

### *TGF-β*_*1*_* polymorphism and concentration of inflammatory markers*

**Fig. 4:**
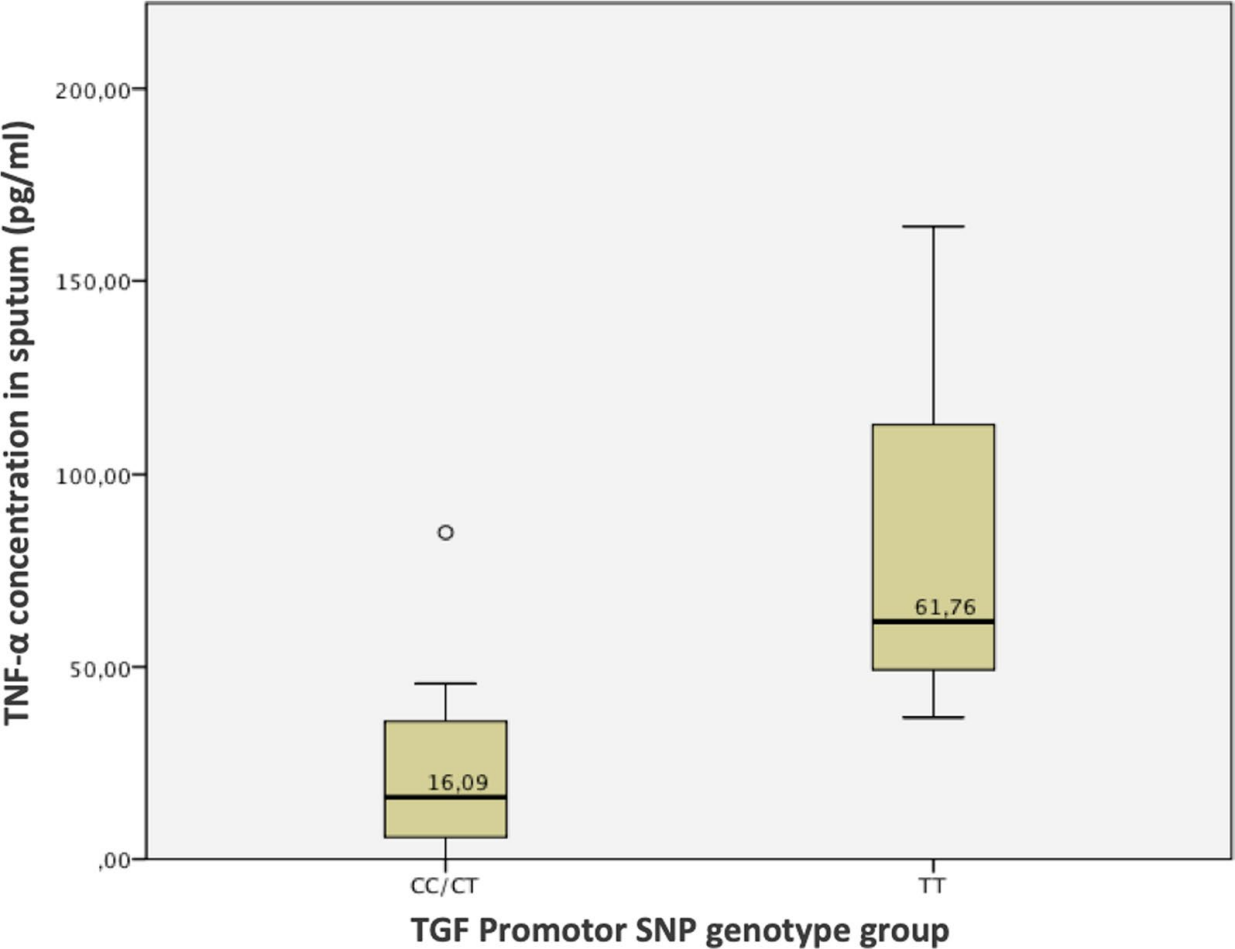
TGF-β_1_ Promotor SNP genotype group and TNF-α concentration in sputum

We found a significantly higher concentration of sputum TNF-α in patients with a homozygous TT genotype at the TGF-β_1_ promotor polymorphism (p = 0.019), as shown in Fig. 4. For all other inflammatory parameters, there was no significant correlation to TGF-β_1_ concentration. A summary of all polymorphisms and corresponding levels of inflammatory markers are listed in Additional file 1: Figure E.

## Discussion

The aim of this study was to investigate whether TGF-β_1_ SNP genotypes, as modifiers of CF lung disease, can be associated with a faster decline in pulmonary function, higher TGF-β_1_ expression, higher *Pa* infection rates and elevated levels of proinflammatory cytokines in sputum. As expected, we found no difference in the genotype frequency distributions of TGF- β_1_ polymorphisms between CF patients and healthy controls. The genotype frequencies for each TGF-β_1_ polymorphism corresponded with known allele frequencies for these polymorphisms as documented following a PubMed dbSNP search [29]. This distribution of the various genotypes demonstrates that the patient cohort is representative for a randomly selected population sample.

We showed that codon 10 TGF- β_1_ polymorphism genotypes (CC or CT) are associated with a higher rate of *Pa* infection (p = 0.047). The observed significant correlation between TGF- β_1_ genotype TT in codon 10 and less chronic *Pa* infection is a finding of special interest. However, it contrasts the findings of Arkwright et al., who found no significant difference between *Pa* infection and TGF- β_1_ codon 10 TT genotype [15]. As shown in studies by Emerson et al. and Thomassen et al., chronic *Pa* infection leads to an activation of profibrotic inflammatory mediators in CF patients and increased pulmonary inflammation resulting in higher mortality rates. [17, 30]. In a recent study by Sagwal et al., increased levels of TGF- β_1_ in serum were found in all types of bacterial infections, with comparably more increase in patients infected with *Pa* [11]. In our cohort, only 16.7% of patients with the homozygous codon 10 TT-genotype were chronically infected with *Pa*, compared to a 42.1% infection-rate in the group of patients with either the CT or CC genotype. Specifically, the high chronic infection rate of 57.1% in the CC group was noticeable at this context. Of all the patients with chronic *Pa* infection in our cohort (n = 19), only 15.2% showed the TT genotype. There might be an unclear mechanism, by which the immunological protection against this pathogen is affected. The noticeably higher infection rate of patients with the CC genotype for this polymorphism might be supported by Drumm et al.’s observed association of this genotype with a worsened lung function, as it also correlates with increased gene expression and circulating TGF- β_1_ levels in their study [13]. Whether TGF- β_1_ with a TT genotype at codon 10 has protective antimicrobial properties, e.g. by contributing to a generally more controlled pulmonary inflammation that significantly lowers the rate of *Pa* infection in this subgroup, or whether the TGF- β_1_ CC-genotype at codon 10 acts as an immunological predisposition for onset of *Pa* infection, is yet to be investigated.

Our results show a significant association between the TGF- β_1_ polymorphism genotypes at the promotor SNP of TGF- β_1_ and the concentration of TNF-α in the patients’ sputum (p = 0,019). Interestingly, the concentration of TNF-α for patients with the homozygous TT promotor genotype at this polymorphism was four times higher than in patients with other genotypes. TNF-α acts as a signal cytokine, that activates the acute phase proteins [31]. Yang et al. hypothesized that colonization with flagellated bacteria, such as *Pa*, may lead to a higher expression of TGF- β_1_ via MAP kinases [32]. In addition to this, Eickmeier et al. found a higher co-expression of TGF-β_1_ and TNF-α in patients with microbiological evidence of at least one type of bacterial infection [3]. In a more recent case–control study by Oueslati et al., CF patients with a TT promoter genotype were associated with worse lung symptoms than patients with other genotypes at this SNP [33]. In summary, our result partially corresponds with the hypothesis of a TGF-β_1_/TNF-α co-expression, as the homozygous TGF-β_1_ promotor genotype TT was significantly associated with a higher expression of TNF-α, despite there being no significant difference in TGF-β_1_ concentration in sputum.

We also found, that the GC genotype of the TGF- β_1_ codon 25 polymorphism correlates significantly with better FEV_1_ slopes in CF patients (p = 0.003). 66.6% of patients with this genotype showed a positive FEV_1_ slope >  + 1% FEV_1_ change/year, compared to merely 10.2% of the patients in the group with homozygous GG genotypes, despite a lower average baseline FEV_1_ in the GC-group. This correlation has not been described previously in other studies before. Although Arkwright’s study on TGF-β_1_ polymorphisms included the combined TT/GG genotype (Codon 10/Codon 25) in the “high-producer” categorization of patients, no correlation was found between codon 25 genotype and measures of survival (age of death/transplantation), *Pa* infection or lung function [15]. In our study, plasma and sputum levels of TGF-β_1_ showed no significant difference between codon 25 genotype groups.

We were able to demonstrate that high TGF-β_1_ plasma levels are associated with a more rapid decline in lung function over a five-year period (p = 0.045). The highest TGF-β_1_ plasma concentrations were found in patients with a more severe FEV_1_ decline over time. This correlation has also been described by Brazova et al. [6] and can be explained by the adverse effects of chronic, systemic inflammation in CF, potentially regulated by TGF-β_1_ in plasma. However, an association of very low TGF-β_1_ concentration with a more deteriorated lung function was also demonstrated in their study, which our results do not confirm.

Interestingly, we found that the highest local TGF-β_1_ concentrations in sputum were associated with intermediate FEV_1_ values (40–80% predicted) of CF patients over 5 years (p = 0.01), whereas for patients with both high (> 80% predicted) or low (< 40% predicted) FEV_1_ values over 5 years, the TGF-β_1_ concentrations were significantly lower. In patients with normal and stable FEV_1_ values above 80% predicted, inflammatory processes might not be activated to the same level as in patients with intermediate FEV_1_ values showing a higher rate of decline. Patients with highly impaired lung function (FEV_1_ < 40% predicted) might show more activation in pulmonary tissue remodeling and pulmonary fibrosis and less active inflammation; this could explain the < 40% FEV_1_ group showing lower TGF-β_1_ concentrations in their sputa. Zemel et al. also showed that an initially high FEV_1_ value in CF patients is linked to worse FEV_1_ progression over time, which may be linked to a less aggressive anti-inflammatory and antimicrobial treatment in children with an initially higher FEV_1_ [34]. This might suggest that the impact of local TGF- β_1_ (in sputum) on pulmonary function is significant, especially in those patients with intermediate FEV_1_ values, when chronic inflammation reaches its maximum during a phase of steady pulmonary function decline of 1–3% FEV_1_/year.

## Limitations of this study

- Despite several statistical comparisons made with SNPs, inflammatory marker concentrations and pulmonary outcomes, we did not conduct a correction for multiple comparisons, as this was an exploratory study.
- The statistically significant correlation between TGF- β_1_ plasma levels vs. FEV_1_ slope, shown in Fig. 2, was not adjusted for baseline differences in FEV_1_ or other markers of disease severity.
- Biomarker concentrations were not transformed prior to analysis to account for possible skew.
- Although FEV_1_ serves as gold standard parameter for evaluation of CF lung disease progression, in patients starting with higher FEV_1_ values (> 80%) small changes in lung function could have been analysed more closely using LCI measurement. This was not performed at the time of the study, as LCI measurement had not yet been established within clinical routine at our CF center at the time of data collection.

## Conclusions

In conclusion our results demonstrate the relevance of the multifunctional cytokine TGF-β_1_ as a genetic modifier in patients with CF. We showed that genetic polymorphisms in the TGF-β_1_ sequence have an impact on pulmonary function, rates of chronic *Pa* infection as well as the concentration of inflammatory cytokines, such as TNF-α. TGF-β_1_ polymorphisms might therefore be used to identify patients with a high risk for disease progression. Furthermore, TGF- β_1_ inhibition could be used as a therapeutic target to prevent the effects of a dysregulated signal pathway leading to higher levels of pulmonary inflammation for certain TGF-β_1_ polymorphisms.

## Supplementary Information

**Additional file 1.** Contains Figures A, B, C, D and E as referred to in the manuscript text above. **Figure A **shows an exemplary capillary electrophoresis result of a CF-patient in our cohort used to determine the genotype at all three investigated TGF-β_1_ polymorphism loci. **Figure B** shows a summary of all FEV_1_slope subgroups and categorizations used for more detailed analysis of slope associations with SNPs and TGF-β_1_ levels. **Figure C** shows a summary of FEV_1_ subgroups, according to best FEV_1_ in the final year of their 5-year observation period. **Figure D** shows a summary of mean average FEV_1_ slope for different SNP genotype groups at all three investigated TGF-β_1_ SNP loci. **Figure E** shows a summary of all TGF-β_1_ SNP genotypes and mean average concentrations of investigated inflammatory markers. 

## Data Availability

The datasets generated and analyzed during the current study are not publicly available due to further studies being conducted with the data. The datasets and materials used are available from the corresponding author on reasonable request. The genetic data of the investigated, previously known TGF-β_1_ polymorphisms can be found under the following web links of the dbSNP database: rs1800469—https://www.ncbi.nlm.nih.gov/snp/rs1800469?horizontal_tab=true. rs1800470—https://www.ncbi.nlm.nih.gov/snp/rs1800470?horizontal_tab=true. rs1800471—https://www.ncbi.nlm.nih.gov/snp/rs1800471?horizontal_tab=true

## Abbreviations

ATS: American thoracic society
A: Adenine
C: Cytosine
CF: Cystic fibrosis
CFTR: Cystic fibrosis transmembrane conductance regulator
ddNTP: Dideoxynucleotide triphosphate
DNA: Deoxyribonucleic acid
FEV_1_: Forced expiratory volume in 1 s
G: Guanine; IL-1β: Interleukine 1 Beta
IL-8: Interleukine 8
IL-6: Interleukine 6
*Pa*: *Pseudomonas aeruginos*a
PCR: Polymerase chain reaction
rs: RefSNP
SAP: Shrimp alkaline phosphate
SBE: Single base extension
SD: Standard deviation
SNP: Single nucleotide polymorphism
T: Thymine
TGF-β_1_: Transforming growth factor beta 1; TNF-α: Tumor necrosis factor alpha
