# Supplementary material for: The effect of TGF-β1 polymorphisms on pulmonary disease progression in patients with cystic fibrosis
Source: BMC Pulm Med. 2022 May 7;22:183. doi: 10.1186/s12890-022-01977-1 (PMC9080196; doi:10.1186/s12890-022-01977-1)
Supplement: Supplementary file 1 — Additional file 1. Contains Figures A, B, C, D and E as referred to in the manuscript text above. Figure A shows an exemplary capillary electrophoresis result of a CF-patient in our cohort used to determine the genotype at all three investigated TGF-β1 polymorphism loci. Figure B shows a summary of all FEV1slope subgroups and categorizations used for more detailed analysis of slope associations with SNPs and TGF-β1 levels. Figure C shows a summary of FEV1 subgroups, according to best FEV1 in the final year of their 5-year observation period. Figure D shows a summary of mean average FEV1 slope for different SNP genotype groups at all three investigated TGF-β1 SNP loci. Figure E shows a summary of all TGF-β1 SNP genotypes and mean average concentrations of investigated inflammatory markers. [file 12890_2022_1977_MOESM1_ESM.docx]

Supplementary Files to Research Article

**The effect of TGF-β_1_ polymorphisms on pulmonary disease progression in patients with cystic fibrosis**


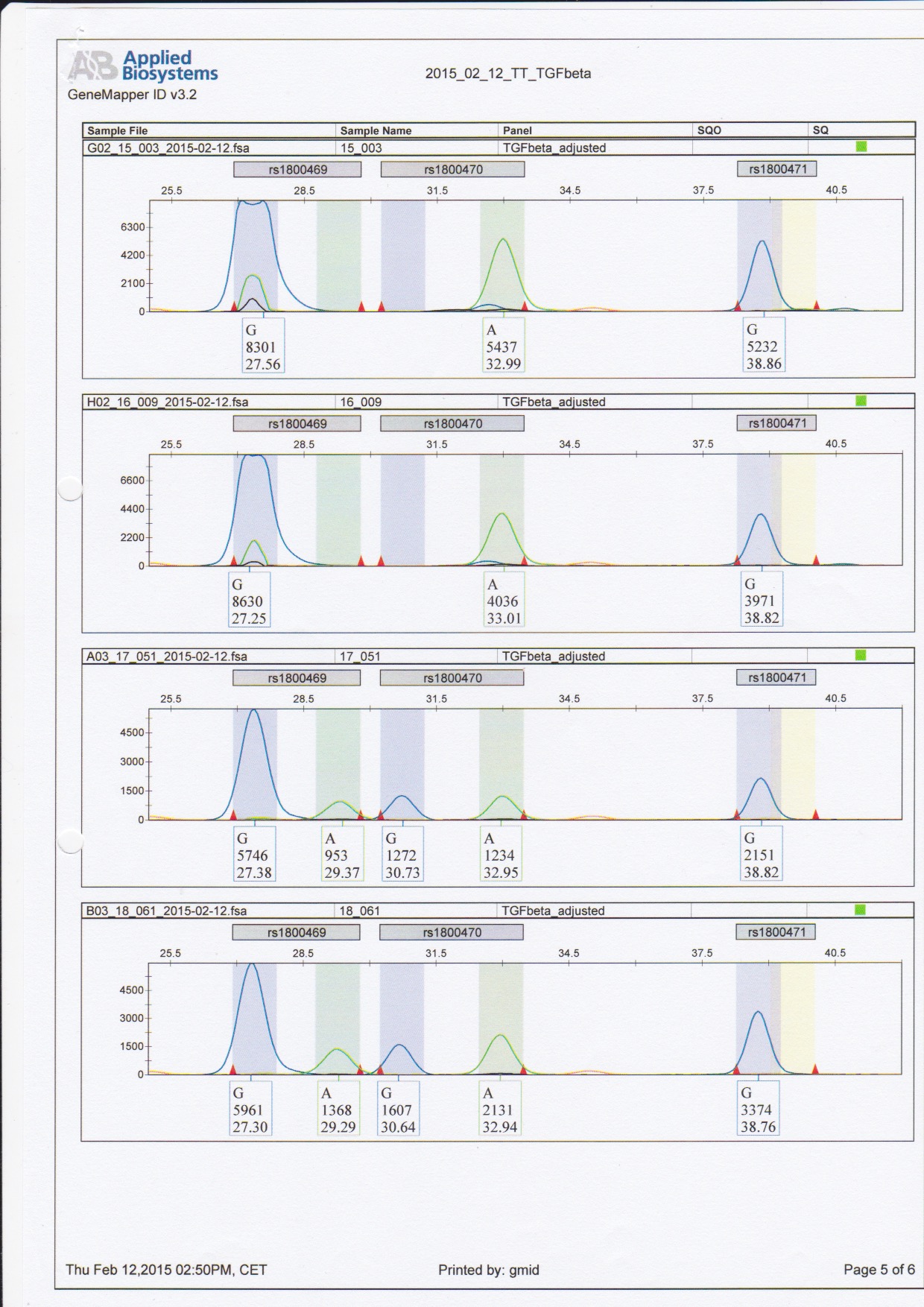
**Figure A**

Exemplary capillary electrophoresis result of CF-patient showing binding of fluorescent bases to sense DNA strand for promotor and Codon 10 genotyping and binding to antisense DNA strand for Codon 25 genotyping. (Promotor genotype = CT, Codon 10 genotype = CT, Codon 25 genotype = GG).

**Figure B**

| **FEV_1_ slope Categorization** | **FEV_1_ Slope Sub-Groups in category**  **[% FEV_1_ change/year]** |
| --- | --- |
| 1 | <0 |
|  | ≥0 |
| 2 | < -1 |
|  | -1 till +1 |
|  | ≥ +1 |
| 3 | < -2 |
|  | -2 till +2 |
|  | ≥ +2 |

Summary of all slope subgroups and categorizations used for more detailed statistical analysis of slope associations with SNPs and TGF-beta1 levels.

**Figure C**

| **Lung function rating** | **Absolute FEV_1_ at the end of 5-year period**  **[% FEV_1_ predicted]** |
| --- | --- |
| Normal | ≥80% |
| Intermediate | 40-80% |
| Low | 40% |

Summary of FEV1 subgroups, according to best FEV_1_ in final year of 5-year observation period.

**Figure D**

| **TGF-β_1_ SNP locus** | **SNP Genotype** | | **Mean FEV_1_ Slope**  **[% change/year]** |
| --- | --- | --- | --- |
|  |  | **n** |  |
| Promotor | CC | 20 | -2,21 |
|  | CT | 31 | -1,51 |
|  | TT | 5 | -2,05 |
| Codon 10 | CC | 6 | -1,70 |
|  | CT | 29 | -1,60 |
|  | TT | 18 | -2,20 |
| Codon 25 | GC | 6 | -1,06 |
|  | GG | 50 | -1,90 |

Mean average FEV_1_ slope for different SNP genotype groups at all three investigated TGF-beta1 SNP loci.

**Figure E**

| **TGF-β_1_ SNP locus** | **Genotype** | **Inflammatory Markers** | | | | | | | |
| --- | --- | --- | --- | --- | --- | --- | --- | --- | --- |
|  |  | **TGF-β_1_ in plasma**  **[pg/ml]** | **TGF-β_1_ in sputum**  **[pg/ml]** | **TNF-α in sputum**  **[pg/ml]** | **Elastase in sputum**  **[pg/ml]** | **Elafin in sputum**  **[pg/ml]** | **IL-1β in sputum**  **[pg/ml]** | **IL-8 in sputum**  **[pg/ml]** | **IL-6 in sputum**  **[pg/ml]** |
| Promotor | CC | 22.2 x 10^3^ | 89,73 | 16,83 | 216,45 | 14.477 | 1.412 | 2.988 | 38,4 |
|  | CT | 16.5 x 10^3^ | 126,48 | 23,93 | 248,42 | 11.317 | 2.004 | 4.320 | 55,5 |
|  | TT | 21.3 x 10^3^ | 69,50 | 87,57 | 362,40 | 20.175 | 2.245 | 3.649 | 51,38 |
| Codon 10 | CC | 16.9 x 10^3^ | 95,50 | 61,50 | 340,47 | 19.182 | 2.232 | 4.078 | 47,01 |
|  | CT | 18.9 x 10^3^ | 126,50 | 23,00 | 245,39 | 11.464 | 2.153 | 4.275 | 57,14 |
|  | TT | 19.7 x 10^3^ | 77,0 | 16,52 | 209,88 | 13.941 | 924 | 2.649 | 30,95 |
| Codon 25 | GC | 21.0 x 10^3^ | 149,17 | 22,23 | 238,65 | 14.445 | 2.760 | 4.349 | 49,13 |
|  | GG | 18.7 x 10^3^ | 103,60 | 30,13 | 251,22 | 13.148 | 1.699 | 3.728 | 49,31 |

**Figure E:** Summary of all genotypes and mean average concentrations of investigated inflammatory markers. As inflammatory marker determination was not possible for all patients, the number of patients (n), for which a value was measurable, is reported as follows: TGF-β_1 Plasma_ (n=54), TGF-β_1 Sputum_ (n = 46), Elastase (n=48), Elafin (n=51), IL-1β (n=48), IL-8 (n=48), IL-6 (n = 39).
